# Increasing physical activity among young children from disadvantaged communities: study protocol of a group randomised controlled effectiveness trial

**DOI:** 10.1186/s12889-016-3743-0

**Published:** 2016-10-19

**Authors:** Rebecca M. Stanley, Rachel A. Jones, Dylan P. Cliff, Stewart G. Trost, Donna Berthelsen, Jo Salmon, Marijka Batterham, Simon Eckermann, John J. Reilly, Ngiare Brown, Karen J. Mickle, Steven J. Howard, Trina Hinkley, Xanne Janssen, Paul Chandler, Penny Cross, Fay Gowers, Anthony D. Okely

**Affiliations:** 1 Early Start Research Institute, Faculty of Social Sciences, University of Wollongong, Northfields Ave, Wollongong, New South Wales 2522 Australia; 2 Institute of Health and Biomedical Innovation at Queensland Centre for Children’s Health Research, School of Exercise and Nutrition Science, Queensland University of Technology, Brisbane, Australia; 3 School of Early Childhood, Queensland University of Technology, Brisbane, Australia; 4 Institute for Physical Activity and Nutrition (IPAN), School of Exercise and Nutrition Sciences, Deakin University, Geelong, Australia; 5 School of Psychological Science and Health, University of Strathclyde, Glasgow, Scotland UK; 6 Institute of Sport, Exercise and Active Living, College of Sport and Exercise Science, Victoria University, Melbourne, Australia

**Keywords:** Preschool, Early years, Physical activity, Motor skill, Professional development, Cluster randomised controlled trial

## Abstract

**Background:**

Participation in regular physical activity (PA) during the early years helps children achieve healthy body weight and can substantially improve motor development, bone health, psychosocial health and cognitive development. Despite common assumptions that young children are naturally active, evidence shows that they are insufficiently active for health and developmental benefits. Exploring strategies to increase physical activity in young children is a public health and research priority.

**Methods:**

*Jump Start* is a multi-component, multi-setting PA and gross motor skill intervention for young children aged 3–5 years in disadvantaged areas of New South Wales, Australia. The intervention will be evaluated using a two-arm, parallel group, randomised cluster trial. The *Jump Start* protocol was based on Social Cognitive Theory and includes five components: a structured gross motor skill lesson (Jump In); unstructured outdoor PA and gross motor skill time (Jump Out); energy breaks (Jump Up); activities connecting movement to learning experiences (Jump Through); and a home-based family component to promote PA and gross motor skill (Jump Home). Early childhood education and care centres will be demographically matched and randomised to *Jump Start* (intervention) or usual practice (comparison) group. The intervention group receive *Jump Start* professional development, program resources, monthly newsletters and ongoing intervention support. Outcomes include change in total PA (accelerometers) within centre hours, gross motor skill development (Test of Gross Motor Development-2), weight status (body mass index), bone strength (Sunlight MiniOmni Ultrasound Bone Sonometer), self-regulation (Heads-Toes-Knees-Shoulders, executive function tasks, and proxy-report Temperament and Approaches to learning scales), and educator and parent self-efficacy. Extensive quantitative and qualitative process evaluation and a cost-effectiveness evaluation will be conducted.

**Discussion:**

The *Jump Start* intervention is a unique program to address low levels of PA and gross motor skill proficiency, and support healthy lifestyle behaviours among young children in disadvantaged communities. If shown to be efficacious, the *Jump Start* approach can be expected to have implications for early childhood education and care policies and practices, and ultimately a positive effect on the health and development across the life course.

**Trial registration:**

Australian and New Zealand Clinical Trials Registry No: ACTRN12614000597695, first received: June 5, 2014.

## Background

The early years (defined here as the first five years of life) are a critical time for the development of lifetime healthy behaviours, including physical activity [1, 2]. Regular participation in light physical activity (LPA) and moderate- to vigorous physical activity (MVPA) during the early years has been shown to help young children maintain a healthy body weight, as well as substantially improve motor development, bone health, psychosocial health and cognitive development [2]. Recently, a number of countries, including Canada [3], United States [4], United Kingdom [5] and Australia [6], have developed activity guidelines specifically for children under the age of five years, with most recommending that children should engage in at least 180 min in any activity daily, including both LPA and MVPA, for general health and developmental outcomes [3, 5, 6]. International studies have reported variations in the percentage of young children meeting these guidelines, with approximately 5 % of Australian young children, 84 % of Canadian young children and 100 % of UK children meeting these guidelines. Variation in the estimates of daily physical activity can partly be attributed to differences in measurement and data processing protocols, and sampling differences [7, 8]. Despite these international variations, there is still a consensus for a need to explore strategies to increase and maintain the number of young children who are sufficiently active for health benefits [1, 9–11].

Recent evidence is emerging to show that engagement in physical activity during the early years has critical health and developmental implications that can persist across the life course. It has been shown that engaging in physical activity stimulates neurocognitive processes and promotes children’s capacity to regulate their behavioural actions [12, 13]. In addition, motor skill development has been shown to be a consistent correlate of physical activity [14, 15] and motor skill proficiency can open up opportunities to be active across a range of settings during the early years [16]. A recent systematic review has also shown the benefits of physical activity on weight status with four out of seven studies showing that children who were more active at age 5, had smaller gains in adiposity over time [2]. Engaging in at least 30 min of MVPA a day at age 5 can significantly increase children’s bone strength at ages 8 and 11 [17]. Given the small but growing body of current scientific evidence of the health benefits of physical activity for young children, promotion of physical activity during the early years needs to be a research priority [18].

Recent attention has focused on Early Childhood Education and Care (ECEC) settings as potential locations for reaching and delivering physical activity interventions for promotion of health and developmental outcomes, particularly those serving families in disadvantaged areas [19]. In a recent report from the Organisation for Economic Co-operation and Development (OECD), almost all OECD countries have adopted quality curriculum standards and frameworks for children aged three years and up [20]. These quality standards and frameworks highlight the need to provide physical activity opportunities for children while they are attending ECEC settings [20]. Despite these quality requirements, children accumulate relatively little physical activity in ECEC settings with children spending on average between 6.2 to 15 % of their ECEC day engaged in physical activity [21, 22], which is well below the recommended 25 % [23–26]. A number of studies have shown that lack of sufficient training in physical activity promotion among early childhood educators, a poor balance between structured and unstructured activity, a lack of equipment and resources and limited or no physical activity practices and policies may be contributing to the low activity levels of children at ECEC centres [1, 27–31].

To date, there is limited evidence of the effectiveness of interventions aimed at increasing physical activity levels of young children in ECEC settings [16, 32]. A recent systematic review conducted by Ward et al. [1] identified 19 studies which reported physical activity interventions in ECEC settings with varied levels of effectiveness. Through the evaluation of these studies, Ward et al. [1] provided recommendations for future ECEC-based interventions, including using a formal curriculum on a regular basis to implement structured physical activity, providing a balance between structured and unstructured physical activity opportunities (such as free or self-directed play), providing sufficient amounts of equipment, and providing regular training for educators in how to provide structured physical activity (particularly around integrating physical activity into the academic curriculum). Due to the infancy of ECEC-based physical activity intervention research, there is a need for additional evidence and evaluation of potential effective strategies in these settings. Therefore, this paper provides a description of the protocols and rationale for the *Jump Start* intervention, a randomised controlled trial implemented in ECEC settings in Australia.

The primary hypothesis is that at post-intervention (18-months), children in ECEC centres allocated to the *Jump Start* intervention will significantly increase total physical activity (i.e. light, moderate, and vigorous intensity physical activity (LMVPA)) while at the ECEC centre compared to children in centres allocated to the usual practice comparison group. The secondary aims are to: (1) examine the relative effects of the intervention on other health and developmental outcomes, including MVPA, sedentary time, self-regulation, weight status, bone strength, and gross motor skills; and (2) explore the potential mediating and moderating variables, cost-effectiveness and implications for public policy decision making.

## Methods

### Study design

**Fig. 1:**
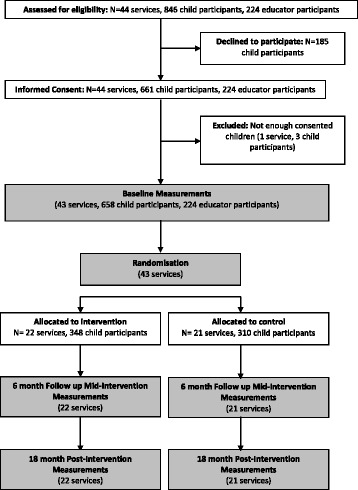
CONSORT flowchart

The *Jump Start* study is a two-arm, parallel group, cluster randomised controlled trial (RCT) using a nested-cohort design [33] (Fig. 1). The development of the *Jump Start* intervention was informed by formative research, involving two ECEC parent organisations and ECEC educators employed by those organisations. The two ECEC parent organisations have collaborated on several of the *Jump Start* pilot studies and provided input into the intervention components and their implementation. Educator professional development and training around physical activity promotion and gross motor development, and focusing on disadvantaged communities were identified by all parties as the highest priorities.

The *Jump Start* intervention has been designed using the recommended framework for developing and evaluating complex interventions [34]. This design enables the assessment of intervention effects at 6- and 18-month follow-up when the children assessed at baseline will still be attending the ECEC centre (i.e., not transitioned to primary school). Assessments took place at baseline (February-June 2015), 6-months (August-December 2015) and will be repeated at 18-months (August–December 2016). The primary outcome is change in total physical activity (LMVPA) while at the ECEC centre over the 18-month study period.

ECEC centres were randomised to either the *Jump Start* intervention or usual practice comparison group. The *Jump Start* intervention is a multi-component, multi-setting approach that combines evidence-based intervention components [28, 30, 31, 35, 36] targeting multiple influences on physical activity among preschoolers (children aged 3–5 years) living in disadvantaged areas of New South Wales (NSW). It consists of five main components: a structured gross motor lesson (Jump In); unstructured outdoor physical activity and gross motor time (Jump Out); energy breaks (Jump Up); activities connecting movement to learning experiences (Jump Through); and a home-based family component to promote physical activity and gross motor development (Jump Home). These components have previously been evaluated independently and have shown high feasibility and potential efficacy across a number of settings [28, 30, 31, 35, 36] but have yet to be tested as a comprehensive approach within ECEC settings.

The trial is being conducted in accordance with Consolidated Standards of Reporting Trials (CONSORT) guidelines for cluster randomised trials [37]. All study procedures have been approved by the University of Wollongong Human Research Ethics Committee (HE14/137) and registered with the Australian and New Zealand Clinical Trials Registry (ACTRN12614000597695).

### Participant recruitment and eligibility criteria

The sampling frame comprised ECEC centres located in areas of disadvantage in NSW, Australia, according to the area location indices for socio-economic disadvantage (SEIFA) [38]. Centres were eligible if they were located in an area with a SEIFA index of relative socioeconomic disadvantage of less than or equal to 5 (lowest 50 %) [38] and had a minimum enrolment of five eligible consenting children. From the 74 eligible ECEC centres, 57 centres were approached and 44 centres were successfully recruited. One centre withdrew from the study due to not having the minimum number of consenting children (*n* = 3).

Children were eligible to participate in the evaluation components of the study if they were 3 years old, or turning 3 before the start of the intervention; attended at least 2 days a week at a participating ECEC centre; and were not likely to be enrolled in primary school the following year. All parents/caregivers of eligible children received a participant information sheet and a URL YouTube link to a recruitment video and were invited to provide written consent to participate. All educators working with 3 year olds in the ECEC centres were also invited to take part in the study and provide written consent to participation. Educators who are employed in centre-based ECEC services across Australia, and who are responsible for a group of children, are required by national regulations to hold at least a 12-month vocational qualification to work with children or be enrolled to complete such a vocational qualification [39]. Recruitment began in January 2015 and was completed in June 2015.

### Power and sample size

Sample size and power estimates were based on the formula proposed by Murray [33] to adjust for a clustered (nested) cohort design. It is recommended that sample size estimates be based on the *a priori* minimum acceptable difference between groups to be considered meaningful [40–42]. Based on our experience and the changes observed in our pilot studies for accelerometer-based physical activity, we estimated this minimum acceptable difference to be 45 mins/day of total physical activity, which translates to an effect size (Cohen’s d) of 0.4. The 6-month Jump Start pilot [14] resulted in a Cohen’s *d* = 0.4 for counts per min, and the 6-month Jump Start translational pilot resulted in a Cohen’s *d* = 0.39 for percentage of time spent in LPA [43]. We therefore concluded that achieving an effect size ≥ 0.4 was realistic and our multi-component intervention strategies, with an increased intervention length and intensity compared to our pilot studies, would be expected to produce an effect size similar to or greater than these studies. For a two-tailed alpha = 0.05 and an intraclass correlation (ICC) of 0.01–0.05 our proposed sample size of 608 participants (304 per group) provides approximately 86 % power to detect an intervention effect of 0.4 or greater for the ICC range proposed.

### Randomisation

Following recruitment and baseline testing, centres were pair-matched according to the number of educators and children in attendance, geographical location and Indigenous status of the centre. Centres within each pair were then randomised to the *Jump Start* intervention or usual practice comparison group by a statistician, not involved in the recruitment or intervention delivery, using a concealed computerised random number generator. The statistician communicated the allocation to the Project Manager, who informed each ECEC centre. Recruitment and baseline assessments were conducted prior to randomisation by trained data collectors blinded to group allocation. The 18-month assessments will also be conducted by trained data collectors blinded to group allocation. To ensure all data collectors remain blinded during the assessment periods, a number of strategies have been put in place to minimise the risk of bias on treatment effect. All data collectors are blinded to study outcomes and hypotheses, and conduct assessments either in the intervention centres only or the usual practice comparison centres only. Educators have been asked not to discuss group allocation with data collectors. In addition, the primary outcome measure and methods, and where possible secondary outcome measurement methods, have been chosen to be as objective as possible in minimising potential for bias. Data collectors are only required to fit a monitor to participants for physical activity assessment, and an external trained assessor, blinded to group allocation, will code videos of the gross motor skills assessments.

### Theoretical framework for the intervention

The *Jump Start* intervention is based on Bandura’s Social Cognitive Theory [44], which has been used extensively in behaviour change interventions. Social Cognitive Theory posits that behaviour is learned, modified and sustained through the interplay of personal, behavioural and environmental factors. The intervention focuses on each factor and how they influence participation in physical activity. To address personal factors, the intervention seeks to increase the emphasis and valuing of children’s physical activity and motor skill development by ECEC educators and parents. To address behavioural factors, there will be a focus on developmentally appropriate activities that build behavioural and motor skills. Educators and parents will have some choice about how they implement some of the components, giving them ownership (control) over children’s learning and the scheduling of intervention sessions at the ECEC centre. The intervention will provide opportunities for educators to set developmentally appropriate mini-goals and provide a sense of achievement. Behavioural skills include goal-setting and self-monitoring of implementation by the educators and parents. Environmental factors will be addressed at both social and physical levels. The social level will incorporate the aforementioned values systems and interaction skills, and will include modifying existing policies and schedules, and using educators to model and reinforce positive attitudes towards physical activity and correct techniques for performing the motor skills. At a physical level, strategies include increasing access and availability to resources that will promote motor development and physical activity in structured and unstructured sessions.

The intervention focuses on the four processes suggested by Bandura [45] to enhance behaviour change (attention, retention, production and motivation). All components of the *Jump Start* intervention have been designed to address all four by: (a) including stimulus material and specific lesson activities that will engage and direct the attention of the educators, parents and children; (b) matching their cognitive and behavioural skill levels in content and pedagogy, and providing opportunities to enhance mastery experiences; (c) including incentives that are relevant, attractive and specified before the learning activities; and (d) emphasising perceived choice and control, as well as challenge, curiosity and mastery through activities that enhance intrinsic motivation, greater persistence, and higher satisfaction [46]. The intervention has been designed to target Social Cognitive Theory mediators which have been identified as mechanisms of behaviour change in physical activity interventions among educators and young children.

### Jump start intervention development

**Fig. 2:**
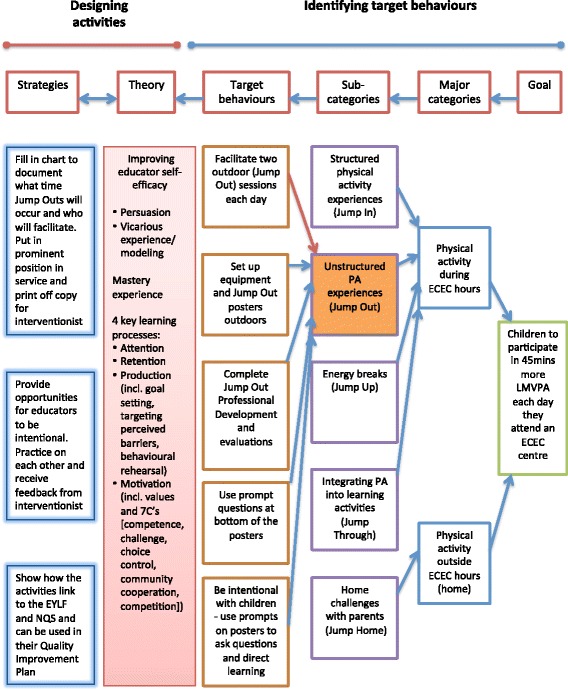
Intervention mapping diagram for the Jump Out component of the *Jump Start* intervention. Similar mapping diagrams have been developed for each of the Jump Start components

An intervention mapping exercise was conducted using the “working backwards” process developed by Robinson and reported in previous interventions [46, 47]. An example of this process for the Jump Out component of the intervention is found in Fig. 2. Briefly, this involves identifying the ultimate goal, mapping all the potential mediating pathways (referred to as Major and Sub-categories in the Figure), and identifying the individual behaviours at the origin of each causal chain of events. Following this, a theoretical framework is applied to develop the *Jump Start* intervention (in this case, Social Cognitive Theory [44]). Specifically, principles of Social Cognitive Theory were applied to generate potential intervention strategies (see far left-hand column in Fig. 2) for enhancing a specific aspect of the theory. For example, for increasing the value that educators place on gross motor skills and physical activity in young children in their Centre, a strategy would be to show educators how the *Jump Start* intervention links to important compliance and curriculum documents such as the Early Years Learning Framework and National Quality Standard [19, 48]. Included in this step is anticipating potential barriers educators may encounter and strategies they could adopt to overcome these barriers. The next step was to evaluate the completed intervention plan (as per Fig. 2) to check if opportunities were provided for the four key learning processes of attention, retention, production and motivation in every element of the *Jump Start* intervention. We also applied additional principles of intrinsic motivation developed by Lepper et al. [49], including competence, challenge, curiosity, control, context, cooperation and competition (referred to as the 7C’s by Robinson [47]), to ensure the messages and activities promoted intrinsic motivation of the targeted behaviours.

Table 1 details the specific components of the *Jump Start* intervention. The *Jump Start* intervention has been contextualised for the ECEC settings by linking it to the sector’s frameworks and curricula (NQS and EYLF) [19, 48] and using sector-specific terminology. All resources and equipment needed to deliver the *Jump Start* intervention will be provided to intervention centres.

**Table 1:** A description of the five components of the *Jump Start* intervention

Jump start component	Description of component	Who facilitates the component
Jump In	Structured gross motor lessons, which will be facilitated every day for approximately 20 min. This component focuses on one gross motor skill, across two lessons every fortnight for 13 skills. All skill lessons are repeated three times over the 18-month period. The skill experiences are based on fun, interactive and engaging games [28].	Educators
Jump Out	Provision of opportunities for children to practise the gross motor skills taught in the Jump In component every day. It provides opportunities for educators to engage with the children in physical activity and encourage the correct performance of the skills. Jump Out is predominantly child-led and educators respond to the child’s cues using a variety of intentional teaching methods.	Educators
Jump Up	Music-based activities designed to break up long periods of sedentary behaviour with high-energy physical activity. The children and educators will engage in two 3-minutes songs every day.	Educators
Jump Through	Activities designed to connect learning and movement [31]. This component aims to use movement to enhance the learning experience. This component will be facilitated twice a day using a range of fun and engaging strategies.	Educators
Jump Home	Opportunities provided to families to learn about Jump Start and for parents/caregivers to participate in the same activities at home that the children have been participating at the ECEC centre.	Parents/caregivers

### Educator training for the intervention

The *Jump Start* intervention is designed to be implemented by ECEC educators. Professional learning was delivered by a trained ECEC educator and provided to other educators through an intensive one-day professional learning session, as well as ongoing bespoke professional learning opportunities provided during the intervention period. An ECEC educator is ideally suited to deliver the professional learning as they have experience working with children in these settings and understand the day-to-day running of an ECEC centre [39]. The one-day intensive professional learning involved 6–8 h of face-to-face contact or virtual contact through teleconferencing technology, and covered background information and the philosophy behind the *Jump Start* intervention, reflection on current practices, content related to each component, opportunities to experience and practice delivery of each component, and a final reflection on how the *Jump Start* intervention could be integrated in the daily routines at the ECEC centres. Free on-going bespoke professional learning was also available to all educators, which focused on additional training in the specific components of the intervention. This additional training was conducted at face-to-face support visits or during the monthly support phone calls provided to all intervention centres.

### Comparison condition

The *Jump Start* intervention is being compared incremental to current usual practice in the ECEC sector. This includes the availability of resources from the *Munch and Move* healthy eating and gross motor skills program [50], which is freely available to all ECEC centres. *Munch and Move* offers online professional learning and support through health promotion officers from the local area health service.

### Strategies to limit attrition

Based on previous feasibility, acceptability and pilot studies of the *Jump Start* components [28, 30, 31, 35, 36], a number of intervention strategies will be implemented to limit attrition. Strategies include: face-to-face support visits and monthly phone calls with all *Jump Start* intervention centres, monthly newsletters providing activity ideas and support information, providing all intervention equipment and resources at no-cost to the *Jump Start* intervention centres, providing non-monetary incentives to the children (e.g. stickers) for completing assessment tasks.

### Outcome measures

Table 2 summarises the outcome measures assessed with children, educators and parents/caregivers. All outcome measures for children were assessed on-site at the ECEC centres.

**Table 2:** Summary of the outcome measures assessed

Outcome measure	Source	Baseline	6-months	18-months
*Children*				
Physical activity^a^	Accelerometry	X	X	X
Gross motor skills	TGMD-2	X		X
Weight status	BMI	X		X
Bone strength	Sunlight MiniOmni Ultrasound Bone Sonometer	X		X
Self-regulation (including executive functioning)	Card Sort task	X		X
	Go No Go task	X		X
	Mr Ant task	X		X
	Not This task	X		X
	Heads-Toes-Knees-Shoulder	X		X
Educator- and Parent-proxy report of children’s approaches to learning	Approaches to Learning scale [58]	X		X
Educator- and Parent-proxy report of children’s temperament	Temperament scale [59]	X		X
*Educators*				
Self-efficacy	Purposively-developed	X		X
Demographics	Purposively-developed	X		
*Parents/caregivers*				
Self-efficacy	Modified questionnaire [61]	X		X
Demographics (including child’s demographics)	Purposively-developed	X		

^a^primary outcome variable; *TGMD-2* Test of gross motor development 2, *BMI* body mass index

#### Primary outcome measure

##### LMVPA while at the ECEC centre

The primary outcome is time spent in total physical activity (LMVPA) while at the ECEC centre as measured by the ActiGraph accelerometer (ActiGraph Corporation, Pensacola, FL), which has established validity and reliability in young children [7, 51, 52]. Children will be asked to wear an accelerometer for 1 week during waking hours, except during water-based activities, at baseline, and at 6- and 18-months. ActiGraph models used in this project will include GT1M, GT3X, and GT3X+, which display high levels of agreement [53]. Collected accelerometer data will be integrated into 15 s epochs during data reduction. After screening for non-wear periods (≥20 min of consecutive ‘0’ counts), participant data will be considered valid at each time point if they accumulate ≥ 3 h of valid wear time during ECEC centre hours on ≥1 ECEC day. These criteria were chosen because: i) 3 h represented 50 % of a typical ECEC day (9 am - 3 pm), and ii) this study is a group RCT and, as such, the aim is to represent LMVPA at the centre level from individual participant samples. Therefore, less stringent inclusion criteria (e.g., ≥ 1 day) is acceptable because these errors may not bias centre-level estimates, and loss of precision may be overcome by increasing sample size. Epochs recording ≥200 counts/15 s will be classified as LMVPA [54].

#### Secondary outcome measures

##### Physical activity and sedentary behaviour while at the ECEC centre

ActiGraph accelerometer data will be used to calculate children’s time spent in moderate (420–841 counts/15 s), vigorous (≥842 counts/15 s) and moderate-to-vigorous physical activity (MVPA) (≥420 counts/15 s), low light-intensity physical activity (26–199 counts/15 s), and sedentary behaviour (≤25 counts/15 s) during ECEC hours using cut-points that have been shown to be most accurate in this age group [51, 52]. Children’s average physical activity (mean activity counts per 15 s) will also be derived.

##### Habitual physical activity and sedentary behaviour

Children’s habitual (during and outside of ECEC hours) physical activity and sedentary behaviour will also be assessed using accelerometry. Children’s data will be included in analyses if they accumulate ≥6 h of valid wear time [55] on ≥1 day.

##### Motor skill development

Gross motor skills will be assessed using the second edition of the Test of Gross Motor Development (TGMD-2) [56] at baseline and 18-months. The gross motor skills assessed include 7 locomotor skills (run, gallop, hop, leap, horizontal jump, slide and balance) and 6 object control skills (striking a stationary ball, stationary dribble, catch, kick, overhand throw and underhand roll). Trained data collectors will follow the TGMD-2 protocols for demonstrating the 13 gross motor skills to the children and use video to capture the children doing the motor skill. The data collectors will not be scoring the children’s gross motor skills. Rather, a trained external blind assessor will score the children’s gross motor skill development using the video footage taken by the data collectors [56]. Each performance criterion within each motor skill will be scored as either a failed attempt or successful completion. Inter-rater reliability will be assessed on a sub-sample of 10 % and acceptable reliability was defined as ICC ≥ 0.70.

##### Weight status

Weight status will be evaluated by measuring height and weight and calculating body mass index (raw BMI scores, weight [kg]/height[m]^2^). Height and weight will be measured at baseline and 18-months by trained data collectors following standardised protocols. Children will be asked to remove footwear and hair adornments (except religious head wear). Height will be measured to the nearest 0.1 cm using stretch stature protocols and a portable stadiometer (SECA 254). Weight will be measured to the nearest 0.1 kg using portable scales (SECA 254). Height and weight measures will be recorded twice and the average of the two measures will be reported. If the two measures differ by more than 0.5 cm for height and 0.5 kg for weight, a third measure will be recorded. Inter-observer reliability will also be assessed on 10 % of the sample. Two measures for both height and weight will be taken on the same participant by a data collector and an independent expert observer. Measurements are required to be within 2 % of an independent expert observer’s measurements.

##### Bone strength

Bone strength will be assessed using the quantitative trans-axial ultrasound method (Sunlight MiniOmni Ultrasound Bone Sonometer) [57] at baseline and 18-months. The MiniOmni Ultrasound Bone Sonometer measures bone speed of sound (SOS; meters per second [m/sec]) using technology based on well-established laws of physics applied to the transmission signals along the bone [58]. After daily System Quality Verification, bone SOS will be measured along the left Tibial crest while the child is seated using a standardised protocol [59], whereby higher SOS values represent greater bone strength. Inter-observer reliability will be assessed by comparing the measurement from the data collector and a qualified biomechanist. Acceptable reliability is defined as a coefficient of variation <0.45 %. The outcome will be reported as a z-score involving units of standard deviations relative to age and gender matched population reference values.

##### Self-regulation

Self-regulation, including executive functioning, will be assessed using a battery of assessments tasks. Children will complete the Head-Toes-Knees-Shoulder task [57]. This measure of behavioural self-regulation requires skills to listen and remember instructions, initiate and stop actions, and sustain attention. Executive function is measured by the Card Sort, Go No Go, Mr Ant and Not This tasks, from the Early Years Toolbox [60]. These tasks measure inhibitory control, working memory and cognitive flexibility. Parents/caregivers and ECEC educators will also report on children’s self-regulation skills, using the 6-item Approaches to Learning scale [58] and an 8-item Temperament scale [59]. The items on these scales capture aspects of children’s capacities for emotional and cognitive control. Children self-regulation skills with this battery of measures will be assessed at baseline and 18-months after intervention commencement.

##### Educator and parental self-efficacy

Educator self-efficacy will be assessed using a purposively developed 12-item questionnaire at baseline and 18-months. This questionnaire assesses educator’s self-efficacy in providing opportunities for physical activity and teaching children gross motor skills. Parental self-efficacy will be assessed using a modified 6-item questionnaire at baseline and 18-months [61]. This questionnaire assesses parent/caregiver’s self-efficacy in providing opportunities for physical activity and teaching children gross motor skills.

##### Demographic characteristics

Demographic information will be collected on the educators, parents/caregivers and the participating children using a questionnaire. Demographic variables include children’s date of birth, sex, Aboriginal or Torres Strait Islander (ATSI) status, and Cultural and Linguistic Diversity (CALD); parent/caregivers’ age, sex, postcode, marital status, education status, employment status, gross annual income, ATSI status, CALD and family structure; and educators’ age, sex, qualifications, years of experience (in ECEC and in the participating centre), and level of training and experience in physical activity and motor skill development. Socio-economic status (SES) is based on postcode of child residence using the Australian Bureau of Statistics census-based SEIFA scores [38].

##### Cost effectiveness analysis measures

Resource use and costs will be collected in the Jump Start and usual care comparison control arms, to determine within study and modelled beyond study incremental costs, effects and cost effectiveness. Resource use and costs of the *Jump Start* intervention will include professional learning trainer and educator time, costed at the relevant wage rates (including penalty rates and on-costs), for one-day *Jump Start* training and implementation time for *Jump Start* with ongoing booster professional learning sessions. Total training time and costs over the study period for the Jump Start and usual care centre educators will be compared to assess the incremental cost of time associated with training, allowing for potential substitution between Jump Start and other training in practice. Any difference in the costs associated with *Munch and Move* health officer support time and disposables will also be estimated in the intervention and usual care comparison control arms.

#### Data collector training

All data collectors will participate in a two-day training workshop. The first day will be a classroom training day, which will cover the specific protocols for each outcome measure and the second day will consist of practical training sessions, in which the data collectors practice measuring each of the outcome measures on a group of preschool-aged children. All data collectors will be required to meet pre-determined inter- and intra-observer reliability standards on similar-aged children and will be monitored periodically throughout the data collection to prevent any observer ‘drift’. At baseline and 18 months, 10 % of the sample will be assessed independently to estimate inter-rater reliability.

### Process evaluation

A range of process data will be collected to assess *Jump Start* intervention fidelity, including adherence and quality of intervention implementation, using both qualitative and quantitative methods. Table 3 provides a description of the process measures and how these data will be collected. Informal feedback and a summative report based on the direct observations will be provided to the centres prior to the next direct observation session. Observation data will be presented as a percentage of intended components completed. These data will be used to classify centres into implementation-level groups (i.e. high, medium or low implementation group) and support strategies will be tailored to each group. For example, centres with low levels of implementation will be offered more support, for example more regular follow-up phone calls compared to centres with high levels of implementation.

**Table 3:** Description of the process data collected to assess *Jump Start* intervention fidelity

How	Process data collected	How Often	By Whom
Self-report checklist	Jump In (length of session in minutes, number of lesson components completed) Jump Out (length of session in minutes, use of posters, activities implemented) Jump Up (number of energy breaks completed) Jump Through (number and description of activities completed)	Completed daily	Educator delivering the component
Direct observation by an independent observer	Jump In (length of session in minutes, number of lesson components completed, number of children participating)	Every 6 months	*Jump Start* research staff
	Jump Out (length of session in minutes, use of posters, activities implemented, number of children participating)		
	Jump Up (length of sessions in minutes, number of energy breaks completed, number of children participating)		
	Jump Through (number of activities, activity intensity of activities, description of activities completed, number of children participating)		
Online survey	Barriers and facilitators to intervention implementation Self-reported observations in children’s behaviour Sustainability strategies Satisfaction with intervention components	At 6- and 18-months	Directors and Educators

Direct observations will also be conducted in comparison centres to monitor the *Munch and Move* program and to document any changes within the centres across the intervention period. Independent research observers will observe 1 day every 6 months and document information on any structured physical activity lessons, unstructured physical activity or gross motor experiences, equipment and resources available and used to promote physical activity, intentional energy breaks, daily group time activities and activity levels of the children during these activities, and communication strategies with families regarding physical activity and gross motor experiences. Directors of each comparison centre will also be asked to complete an online survey about any new or existing activities provided to children in the centre that are intervention-like in nature.

### Statistical analysis

#### Primary analysis

Analysis of the primary outcome will be conducted using a linear or generalized mixed model. The mixed model will contain a random effect for time and ECEC centre nested within group. Degrees of freedom will be altered manually in the code to adjust for the effect of clustering. These established procedures are well documented by Murray [33] and have been used previously by the authors to analyse a similar study in primary schools [62]. No interim analyses will be conducted before all data have been collected.

#### Secondary analyses

Mixed models will also be used to analyse the differences between intervention and comparison groups for all continuous secondary outcome variables.

#### Mediation and moderation analyses

Two types of analyses will be conducted to explore the theoretical assumptions of the intervention. First, hypothesised mediators of change in physical activity (e.g., staff self-efficacy and child motor skills) will be examined using multilevel linear analysis and a product-of-coefficients test appropriate for cluster RCTs [63]. Potential moderators of the intervention effects (e.g., child age and sex) will also be explored using multi-level modelling.

#### Per-protocol analyses

A per-protocol or dose–response analysis will also be performed at the centre and child levels. Child-level compliance with measurement of outcomes will be defined as having worn the accelerometers for at least 3 h per preschool day. Centre-level compliance will be defined as: 1) implementing greater than or equal to 90 % of Jump In and Jump Out sessions; and 2) greater than or equal to 80 % of Jump Up activities and Jump Through activities implemented. Although all children and centres will be included in the intention-to-treat analyses, only children and centres that comply with all of the above requirements will be included in the per-protocol analysis. These compliance measures will also be used to determine the relative effectiveness of each intervention component which will be important to guide further translational work.

#### Economic analyses

Over the intervention period, incremental effects on the primary outcome will be compared with incremental costs to inform within-trial cost effectiveness and extended to secondary outcomes where appropriate for cost effectiveness analysis beyond study. Within the trial, incremental costs and effects under uncertainty and their bivariate distribution will be estimated with bootstrapping (re-sampling with replacement) on paired centre level costs and effects (with centre level effects in turn bootstrapping on individual child effects). This approach allows robust estimation of the joint distribution under uncertainty and for the relationship (covariance) between costs and effects observed within the study and the uncertainty of effects across children within centres [64]. Standard summary measures for cost-effectiveness analysis, including net benefit curves, cost-effectiveness acceptability curves, and expected net loss curves and frontiers will be presented to best inform societal decision makers of the net benefit of the intervention and value of future research [65, 66].

## Discussion

This paper presents the study protocols for the *Jump Start* intervention. This 18-month intervention is unique in its approach to supporting the health behaviours of children living in disadvantaged communities, drawing on evidence and lessons learnt from pilot studies [28, 30, 31, 35, 36] and fostering strong collaborative partnerships between the ECEC sector and multi-disciplinary research team. To the authors’ knowledge, *Jump Start* is one of the first interventions that uses a comprehensive multi-component, multi-setting approach to address the low levels of physical activity and gross motor skill proficiency among preschool-aged children in disadvantaged communities [1].

Targeting young children from disadvantaged communities is important because they typically have limited access to physical activity opportunities, and reduced physical activity levels and gross motor skill proficiency [67–69]. Through the *Jump Start* intervention, we expect to increase total physical activity by a minimum of 45 min/day, 20 min of which will be MVPA. We expect that large and meaningful benefits will flow into other areas of health and development, as evidence shows increases of this magnitude are associated with significant declines in adiposity and significant increases in bone health at ages 8 and 11 years [17]. This approach may also improve behavioural self-regulation skills in this age group, which are better predictors of school readiness than IQ or entry-level literacy or numeracy skills [70].

*Jump Start* will also enhance the quality of early childhood education and care at a crucial time in children’s lives when the architecture of the developing brain is most open to the influences of experiences and when health inequality gaps are smallest. Benefits also accrue for parents and educators. *Jump Start* focuses on capacity building and enhancing the knowledge and self-efficacy of all participating ECEC educators in teaching gross motor skills and integrating physical activity into daily routines through targeted professional learning and on-going support.

In addition, the *Jump Start* intervention, if effective in improving physical activity and educational outcomes of disadvantaged children, can reduce social system costs and benefit the economy. While $1 invested in effective education has shown a long term return of $1.50–$3 across populations, a benefit ratio for $1 of effective education in disadvantaged children (the target population of this intervention) has been estimated at $17 [71]. If children enter primary school with greater skills or learning in our focus areas (motor development, physical activity, behavioural self-regulation) they are less likely to repeat grades or drop out of school, and more likely to enrol in post-secondary education [71, 72].

The *Jump Start* intervention is a unique program to address low levels of physical activity and gross motor skill proficiency, and support healthy lifestyle behaviours among young children in disadvantaged communities. If shown to be efficacious, the *Jump Start* approach can be expected to have implications for ECEC policies and practices, and ultimately a positive effect on the health and development across the life course.

## Abbreviations

ATSI: Aboriginal and Torres Strait Islander
BMI: Body mass index
CALD: Cultural and linguistic diversity
CONSORT: Consolidated standards of reporting trials
ECEC: Early childhood education and care
EYLF: Early years learning framework
GMS: Gross motor skills
ICC: Intraclass correlation
LMVPA: Light-moderate-vigorous physical activity
MVPA: Moderate-vigorous physical activity
NQS: National quality standards
OECD: Organisation for economic co-operation and development
PA: Physical activity
RCT: Randomised cluster trial
SD: Standard deviation
SEIFA: Socio-economic indexes for areas
SES: Socioeconomic status
SOS: Speed of sound
TGMD-2: Test of gross motor development
